# Platelet storage duration and its clinical and transfusion outcomes: a systematic review

**DOI:** 10.1186/s13054-018-2114-x

**Published:** 2018-08-05

**Authors:** Cécile Aubron, Andrew W. J. Flint, Yves Ozier, Zoe McQuilten

**Affiliations:** 1 The Medical Intensive Care Unit, Centre Hospitalier et Universitaire de Brest - Université de Bretagne Occidentale, Bvd Tanguy Prigent, 29609 Brest Cedex, France; 2 The Australian and New Zealand Intensive Care Research Centre, Department of Epidemiology and Preventive Medicine, Monash University, Melbourne, Australia; 3 The Transfusion Research Unit, Department of Epidemiology and Preventive Medicine, Monash University, Melbourne, Australia; 4 Royal Australian Navy, Australian Defence Force, Canberra, Australia; 5 The Department of Anesthesiology, Centre Hospitalier et Universitaire de Brest - Université de Bretagne Occidentale, Brest, France

**Keywords:** Platelet storage, Allogeneic transfusion, Critically ill patients, Haematology patients, Complication, Efficacy

## Abstract

**Background:**

Platelets (PLTs) are usually stored for up to 5 days prior to transfusion, although in some blood services the storage period is extended to 7 days. During storage, changes occur in both PLT and storage medium, which may lead to PLT activation and dysfunction. The clinical significance of these changes remains uncertain.

**Methods:**

We performed a systematic review to assess the association between PLT storage time and clinical or transfusion outcomes in patients receiving allogeneic PLT transfusion. We searched studies published in English between January 2000 and July 2017 identified from MEDLINE, Embase, PubMed and the Cochrane Libraries.

**Results:**

Of the 18 studies identified, five included 4719 critically ill patients (trauma, post-cardiac surgery and a heterogeneous population of critically ill patients) and 13 included 8569 haematology patients. The five studies in critically ill patients were retrospective and did not find any association between PLT storage time when PLTs were stored for up to 5 days and mortality. There was also no association between older PLTs and sepsis in the two largest studies (*n* = 4008 patients). Of the 13 studies in haematology patients, seven analysed prolonged storage time up to 6.5 or 7 days. Administration of fresh PLTs (less than 2 or 3 days) was associated with a significant increase in corrected count increment (CCI) compared to older PLTs in seven of the eight studies analysing this outcome. One single centre retrospective study found an increase in bleeding events in patients receiving older PLTs.

**Conclusions:**

PLT storage time does not appear to be associated with clinical outcomes, including bleeding, sepsis or mortality, in critically ill patients or haematology patients. The freshest PLTs (less than 3 days) were associated with a better CCI, although there was no impact on bleeding events, questioning the clinical significance of this association. However, there is an absence of evidence to draw definitive conclusions, especially in critically ill patients.

**Electronic supplementary material:**

The online version of this article (10.1186/s13054-018-2114-x) contains supplementary material, which is available to authorized users.

## Background

Increasing platelet (PLT) demand coupled with their relatively short shelf-life may compromise PLT availability. One possible solution to address increasing demand and improve PLT availability is an extension of PLT storage duration. PLTs are stored at 22 °C to preserve function; however, this temperature facilitates growth of bacterial contaminants. As a result, PLT storage duration is commonly 5 days, although in some countries that screen for bacterial contamination or use pathogen reduction technologies, this duration is up to 7 days [1, 2]. Over time, changes in both PLTs and their storage medium occur, with an accumulation of bioreactive substances. Extension of PLT storage duration may expose patients to potential decreases in PLT transfusion efficacy as well as possible increases in adverse events in addition to transfusion-associated sepsis, such as inflammation and/or immune-mediated events [3–5]. Critically ill patients, including post-cardiac surgery patients, are the second largest group to receive PLTs after haematology/oncology patients and may be particularly susceptible to PLT adverse events due to their pre-transfusion inflammatory state [6, 7]. Nonetheless, the impact on clinical and transfusion outcomes of PLT storage changes is poorly described in this population [7–11].

**Fig. 1:**
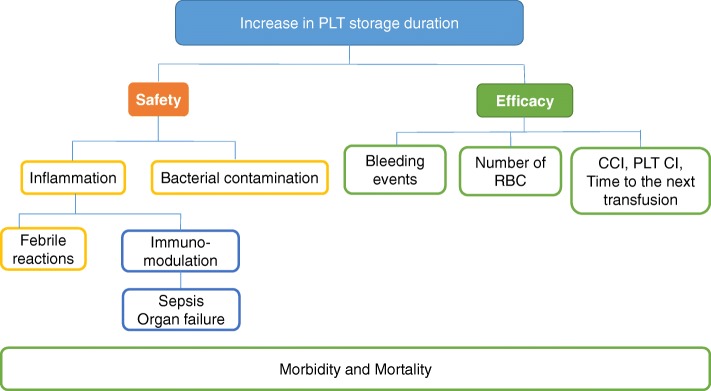
Endpoints to evaluate safety and efficacy of stored PLTs. *RBC* red blood cell, *PLT* platelets, *CCI* corrected count increment, *PLT CI* platelet count increment

The consequences of storage duration on PLT transfusion efficacy can be assessed using transfusion outcomes such as post-transfusion absolute platelet count increment (CI), the corrected count increment (CCI; absolute platelet count increase normalised to body surface area and platelet dose) and the time to next PLT transfusion. More importantly, efficacy can also be assessed using clinical outcomes, including prevention or treatment of bleeding, volume of red blood cells (RBCs) required and mortality. The safety of stored PLTs can be assessed from adverse events following PLT transfusion, such as febrile non-haemolytic transfusion reactions, transfusion-transmitted infection and overall morbidity and mortality (Fig. 1). Difficulties in determining whether stored PLTs are as safe and as effective as fresher PLTs in critically ill patients are related to the fact that most of these outcomes are affected by other factors, including population characteristics, severity of underlying illness, cause of thrombocytopenia, concomitant bleeding, administration of other blood products and other co-morbidities impacting on these endpoints [12].

Some reviews have highlighted the issues around PLT availability, safety of stored PLTs and the potential alternatives to liquid PLTs [13–15]. Two recent meta-analyses have quantified the association between PLT storage duration and PLT measurements after transfusion or clinical outcomes [16, 17]. However, there is a need for a comprehensive systematic review to describe the available literature on the potential association between PLT storage duration and clinical- and transfusion-centred outcomes relevant to the critically ill population, as well as haematology patients—the largest recipients of platelets. Therefore, we conducted a systematic review of the available literature for evidence on the association between prolonged PLT storage and clinical or transfusion outcomes, for both critically ill and haematology patients.

## Methods

This review has been designed to maximise adherence to the Preferred Reporting Items for Systematic reviews and Meta-Analyses (PRISMA) statement [18].

### Eligibility criteria, information sources and search strategy

We systematically searched MEDLINE, Embase and Cochrane databases via OVID and PubMed using keywords (Additional file 1). The searches were restricted to human studies, English language papers only and papers published between January 2000 and July 2017 because PLT processing has changed over the past decades and the results of the less recent studies might not be applicable to current PLT products. We included English language papers only because it was the only consistent language across authors. We included both observational and interventional studies. We considered only available, full-text journal articles and did not include abstracts of oral presentations or posters. We excluded pre-clinical studies and autologous transfusion studies. Identification and selection of the studies was based on the following PICOs (Patients, Interventions, Comparators, Outcomes): Patients, critically ill or haematology patients; Intervention, allogeneic PLT transfusion of “old” PLTs (i.e. PLTs stored for a longer time); Comparator, allogeneic PLT transfusion of “young” PLTs (i.e. PLTs stored for a shorter time); Outcomes, clinical centred outcomes, including mortality, morbidity (infection and length of stay), bleeding and volume of RBCs transfused and transfusion-centred outcomes, including time to next transfusion, rate of successful transfusion based on a PLT CI above a certain cut-off, PLT CI and CCI defined as: [(Post-transfusion (× 10^9^/L) − Pre-transfusion PLT count (× 10^9^/L)) × Body surface area (m^2^)]/Number of transfused PLTs × 10^11^). Eligibility assessment based on the title or abstract and on full text if required was performed independently by two authors (CA and AF). Because a large part of the available evidence comes from studies in haematology patients, and because findings of these studies may apply to critically ill patients, our review research was not restricted to critically ill patients. The review protocol has not been published previously and is not registered on *PROSPERO*.

### Data collection process and study quality assessment

The following were extracted by two authors (CA and AF): study type, sample size, population, PLT manufacturing information if available (including apheresis or pooled), study design (adjustment for confounders, outcome, comparative group) and outcomes. Risk of bias was also assessed using the Cochrane tool [19] and the Risk of Bias in Non-randomised studies of Interventions assessment tool [20]. Discrepancies were discussed and resolved with a third author (ZM) if required. Due to the heterogeneous nature of the studies included with regard to design and outcomes, no quantitative analysis was performed.

## Results

After applying restrictions, 420 results were obtained searching PubMed and 940 results were obtained searching the Cochrane, Embase and Medline databases via OVID.

**Fig. 2:**
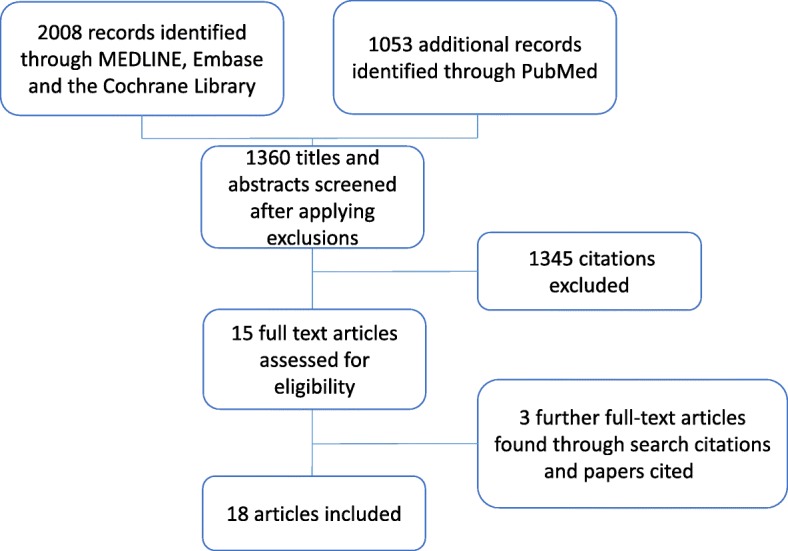
Review flow chart

Results were analysed and either included or excluded based on our inclusion criteria and yielded 15 relevant papers. We further identified three relevant papers through hand searching reference lists and cited articles, giving a total of 18 papers for inclusion in the systematic review (Fig. 2). Five included critically ill patients and 13 haematology patients. Studies addressing the issue of the impact of PLT storage duration on clinical or transfusion outcomes have been classified based on the study population (critically ill patients or haematology patients) and on their outcomes (clinical centred and transfusion centred outcomes). The 18 studies are detailed in Tables 1 and 2.

**Table 1:** Studies analysing the potential association between PLT storage duration and clinical or transfusion outcomes in critically ill patients

Study	Population	Study design	PLT characteristics and maximum storage duration	Primary outcome	Secondary outcome	Analyses/comparator	Main results
Welsby et al. [23]	Cardiac surgery, 2578 patients	Retrospective single centre	Leucoreduced Apheresis 5 days	30-day mortality or hospital stay > 10 days	Infections Survival up to 5 years RBC requirement	All patients Patients with only one unit Oldest PLT transfused	No association
Inaba et al. [21]	Trauma, 381 patients	Retrospective two-centre	Apheresis 5 days	Mortality	Sepsis ARDS AKI Liver failure ICU and hospital LOS RBC requirement	Patients with only PLTs stored for ≤ 3 days, 4 days and 5 days	No association with mortality More sepsis in the group transfused with PLTs stored for 5 days
Juffermans et al. [24]	ICU, 134 patients	Retrospective single centre	Leucoreduced WBD, buffy coat derived ^a^	Nosocomial infections	–	Number of PLTs > 4 days	No association
Juffermans et al. [25]	Trauma, 196 patients	Retrospective single centre	Leucoreduced WBD, buffy coat derived ^a^	Nosocomial infections	–	Number of PLTs > 4 days	No association
Flint et al. [22]	ICU, 1430 patients	Retrospective two-centre	Leucoreduced Apheresis and WBD, buffy coat derived 5 days	Mortality	Blood stream infections and bacteriuria RBC requirement	All patients and patients receiving only one PLY unit Oldest PLTs, freshest PLTs and mean age of all PLTs	No association

*Abbreviations*: *ARDS* acute respiratory distress syndrome, *AKI* acute kidney injury, *PLT CCI* platelet corrected count increment, *ICU* intensive care unit, *LOS* length of stay, *WBD* whole blood derived, *PLT* platelet, *PLT CI* platelet count increment

^a^PLT storage duration not specified

**Table 2:** Studies analysing the potential association between PLT storage duration and clinical or transfusion outcomes in haematology or oncology patients

Study	Population	Study design	PLT characteristics and maximum storage duration	Primary outcome	Secondary outcome	Analysis/comparator	Main results
Van Rhenen et al. [35]	Haematology Prophylactic PLTs 103 patients	Propsective observational study for the review outcome^a^	Leucoreduced WBD, buffy coat derived 5 days	1-h CCI	24-h CCI	Up to 5 days	Association between PLT storage time and 1 and 24-h CCI
Dijkstra-Tiekstra et al. [28]	Haematology Prophylactic PLTs 70 patients, 389 transfusions	Prospective observational single centre	Leucoreduced, WBD, buffy coat derived 7 days	1-h CI ≥ 10 1-h CCI ≥ 7.5	1-h CCI	2, 3, 4, 5, 6 and 7 days	No difference between 5 and 7 days of storage; PLTs stored for 2 days had higher 1-h CCI than PLTs stored for 7 days
Slichter et al. [12]	Haematology 528 patients Prophylactic transfusion	Post hoc analysis of a randomised trial	Apheresis and WBD, plasma rich preparation 5 days	1- and 24-h CCI	Time to next transfusion Refractoriness	2, 3, 4 and 5 days Analysis per transfusion	PLTs stored less than 48 h were associated with better 1- and 24-h CCI and s longer time to next transfusion
Akkok et al. [31]	Haematology 77 patients Prophylactic and therapeutic transfusions	Prospective observational, two-centre	Leucoreduced Apheresis and WBD, buffy coat derived 6.5 days	1-h CCI	18–24 h CCI Time to next transfusion	Up to 6.5 days	Decrease in 1- and 24-h CCI and time to next transfusion when storage time increased
Heim et al. [36]	Haematology 672 patients Prophylactic transfusion	Prospective observational	Apheresis 5 days	1- and 24-h CCI	TRAE	Up to 5 days	PLTs stored for ≥ 3 days were associated with a lower CCI
Diedrich et al. [27]	Haematology 60 patients Prophylactic transfusion	Double blind randomised, single centre	Leucoreduced Blood group O irradiated WBD, buffy coat derived 7 days	1- and 24-h CCI	Time to next PLT transfusion Bleeding	Storage duration:1 to 5 days versus 6 to 7 days Mean storage 2.9 days vs 6.6 days	Higher CCI and longer time to next transfusion in the group “fresher PLT”
Kerkhoffs et al. [32]	Haemato-oncology 278 patients	Prospective multicentre observational study for the review outcomes^a^	Leucoreduced, WBD, buffy coat derived 7 days	1-h CCI	24-h CCI RBC number Bleeding Time to next transfusion	Up to 7 days	Storage time was associated with 1- and 24-h CCI but not with bleeding
Triulzi et al. [34]	Haematology 1231 patients	Post hoc analysis of a multicentre RCT	Apheresis or WBD plasma rich preparation Leucoreduced 5 days	Clinical bleeding (time from first transfusion to first ≥ grade 2 bleeding)	Absolute PLT CI and CCI 4, 16 and 24 h	Age of PLTs of the first transfusion	No association between PLT storage duration and bleeding PLTs stored for ≤ 3 days were associated with higher CCI and PLT CI
Heuft et al. [33]	Haematology 77 patients 526 prophylactic transfusions	Retrospective single centre	Apheresis PLTs 5 days	24-h CCI	Time to next transfusion RBC number Bleeding	Max 4 days versus 3 days Subgroup with only one transfusion/day, without RBC transfusion	Fresher PLTs were associated with higher CCI and less bleeding
Dijkstra-Tiekstra et al. [29]	Onco-haematology 93 patients with only one PLT/day 389 transfusions	Prospective observational, three-centre	WBD, buffy coat derived 7 days	1- and 24-h CCI	–	2, 3, 4, 5, 6, 7 days	No association
MacLennan et al., [26]	Haematology 122 patients	Crossover blocked randomised trial; 2 to 5 days versus 5 to 7 days	Apheresis and WBD buffy coat derived, leucoreduced 7 days	CCI > 4.5 between 8 to 24 h	CCI Time to next transfusion Bleeding events (WHO classification)	Mean age 3.8 days (SD1.0) versus 6.4 (0.5) days	No effect of the age of PLTs on primary outcome and bleeding
Kaufman et al. [4]	Haematology 1102 patients, 5034 transfusions	Post hoc analysis of a multicentre RCT	Apheresis or WBD plasma rich preparation, leukoreduced 5 days	TRAE	TRAE considered separately	0–2 days, 3 days, 4 days versus 5 days as reference	No association
Kaplan et al. [30]	Haematology 146 patients, 242 transfusions	Retrospective single centre study	WBD riboflavin based PRTs 7 days	24-h PLT CI	TRAE	Up to 5 days versus 6–7 days	No association

*Abbreviations*: *CCI* corrected count increment, *WBD* whole blood derived, *RCT* randomised controlled trial, *PAS* platelet additive solution, *PLT* platelet, *PLT CI*, platelet count increment, *PRT* pathogen reduction technology, *SD* standard deviation, *TRAE* transfusion-related adverse events, *WHO* World Health Organisation

^a^These studies are randomised trials, but randomisation was not performed by PLT storage duration; therefore, they are considered as observational studies

### PLT storage and critically ill patients

Five studies analysing the impact of PLT storage duration on clinical outcomes in critically ill patients were identified; no study analysed transfusion-centred outcomes in this population (Table 1). All were retrospective single or two-centre studies, and PLT storage was documented or presumed to be up to 5 [21–23] or 7 days depending on local maximum storage duration [24, 25].

#### Mortality

Of the three studies analysing PLT storage duration and mortality in critically ill patients, none reported an association between hospital, 30-day or 5-year mortality and transfusion of older PLTs [21–23].

#### Length of stay

Two studies analysed the potential association between length of stay and PLT storage time and did not find any association between these variables [21, 23].

#### Infection and morbidity

Of the five studies analysing the risk of infection or sepsis after stored PLT administration, only one found an association between older PLT and sepsis [21–25]. In a retrospective study of 381 trauma patients receiving at least one unit of PLTs, Inaba et al. compared outcomes of patients receiving only PLTs that had been stored for less than 3, 4 and 5 days [21]. Patients receiving PLTs stored for 5 days had more complications due to an increase in sepsis (16.4% had sepsis versus 9.2% in the 4-day storage duration group and 5.5% in the ≤3-day storage duration group, adjusted *p* < 0.03). After adjustment for confounders, transfusion of PLTs stored for 5 days was independently associated with complication occurrence (adjusted odds ratio (OR) 2.4, 95% confidence interval 1.4–4.7, *p* = 0.02), suggesting that PLTs stored for 5 days could be associated with an increased risk of infection. Nonetheless, this study had no clear criteria or definition for sepsis and, therefore, the generalisability of this finding is uncertain and has not been found in subsequent studies. Flint et al. [22] reviewed 1430 critically ill patients transfused with at least one unit of PLTs in the intensive care unit (ICU) of two hospitals. After adjusting for confounders and approaching the age of PLTs in several ways (age of the oldest, of the freshest, median age of all transfused PLTs, and the age of the first unit transfused), considering several subgroup analyses and using a robust definition for infection based on microbiological data, the authors did not find any association between PLT storage duration and infectious risk [22]. Although the three other studies investigating whether stored PLTs increase the risk of infection also suffer limitations in their methods, they did not find any association between transfusion of older PLTs and infections [23].

#### RBC requirement

Although the available studies in critically ill patients did not investigate the association between PLT storage duration and transfusion efficacy, some did report RBC requirement as a surrogate of haemostatic PLT function [21–23]. Inaba et al. did not find any difference in number of PLTs, RBCs or fresh frozen plasma units transfused in 381 trauma patients receiving fresh (≤3 days) versus old (4 days and 5 days) PLTs [21]. Two other studies reported similar results, suggesting that PLT storage duration did not impact on efficacy to treat or prevent bleeding [22, 23].

Based on these available studies, transfusion of older PLTs (up to 5 and 7 days) does not appear to be associated with an increased risk of poor outcome in critically ill patients. However, these findings are limited by the nature of the included studies, including their retrospective design (Table 3), and findings therefore need to be confirmed with prospective research.

**Table 3:** Impact of storage duration on corrected count increment and time to next transfusion in haematology patients

Study	1–24 h CCI	Time to next transfusion (days)
Van Rhenen et al. [35]	Decrease in CCI with increased storage time	–
Dijkstra-Tiekstra et al. [28]	Decrease in CCI with increased storage time	–
Slichter et al. [12]	Decrease in CCI with increased storage time	–
Akkok et al. [31]	Decrease in CCI with increased storage time	Decreased time to next transfusion with decrease in storage time
Heim et al. [36]	Decrease in CCI with increased storage time (hazard ratio 1.201, 95% confidence interval 1.065–1.354, *p* = 0.003)	–
Diedrich et al. [27]	Decrease in CCI with increased storage time (mean 5.4 ± 4.1 versus 2.6 ± 2.6, *p* < 0.001)	Decreased time to next transfusion with decrease in storage time (mean time 2.2 ± 1.1 days versus 1.6 ± 0.8 days, *p* < 0.005)
Kerkhoffs et al. [32]	Decrease in CCI with increased storage time	–
Triulzi et al. [34]	Decrease in CCI with increased storage time	No association
Heuft et al. [33]	Decrease in CCI with increased storage time (median 8.3 [IQR 3.9–13.1] versus 3.5 × [IQR 1.5 10.0], *p* < 0.01)	Decreased time to next transfusion with decrease in storage time (median 1.1 day versus 2 days, *p* < 0.001)
Dijkstra-Tiekstra et al. [29]	Decrease in CCI with increased storage time	–
MacLennan et al., [26]	No association	No association
Kaplan et al. [30]	No association	–

*Abbreviations*: *CCI* corrected count increment, *IQR* interquartile ranges

### PLT storage and haematology patients

In haematology patients, 13 studies have investigated the potential association between storage duration and transfusion or patient outcomes, and one thoroughly analysed the impact of PLT storage duration on transfusion-related adverse events (TRAEs; Table 2) [4].

Of these 13 studies, seven considered PLT storage up to 6.5 or 7 days [26–32], and six analysed PLT storage duration up to 5 days [4, 12, 33–36]. Four studies were randomised controlled trials [26, 27, 32, 35], with two comparing outcomes after transfusion of PLTs stored for different times [26, 27] and two comparing outcomes after PLT transfusions with different characteristics not related to but adjusting for PLT storage time [32, 35], three were post hoc analyses of large randomised trials [4, 12, 34] and six were prospective or retrospective single-centre studies [28–31, 33, 36]. The results of these studies are described subsequently according to the analysed outcomes. Assessment of the risk of bias is summarised in Tables 4 and 5.

**Table 4:** Bias assessment of the randomised controlled trials included in this review

Study	Allocation concealment	Random sequence generation	Blinding (participant, physician)	Blinding (outcome)	Incomplete outcome data	Selective reporting	Other
Diedrich et al. [27]	Low risk	Unclear	Low risk	Low risk	Low risk	Low risk	Similar number excluded due to wrong PLT storage time each arm
MacLennan et al. [26]	Low risk	Low risk	Unclear risk	Low risk	Low risk	Low risk	Off protocol transfusion

**Table 5:** Bias assessment for primary outcomes of included observational studies

Study	Bias due to confounding	Selection bias	Bias due to classification of interventions	Deviation from protocol	Missing data	Measurement of outcomes	Selective reporting	Overall risk of bias
Outcome: mortality								
Welsby et al. [23]	Moderate risk	Low risk	Low risk	Low risk	Low risk	Low risk	Moderate risk	Moderate risk
Inaba et al. [21]	Moderate risk	Low risk	Low risk	Low risk	Unclear risk (no information)	Low to moderate risk	Moderate risk	Moderate risk
Flint et al. [22]	Moderate risk	Low risk	Low risk	Low risk	Low risk	Low risk	Moderate risk	Moderate risk
Outcome: nosocomial infections								
Juffermans et al. [24]	Moderate risk	Low risk	Low risk	Low risk	Unclear risk (no information)	Low risk	Moderate risk	Moderate risk
Juffermans et al. [25]	Moderate risk	Low risk	Low risk	Low risk	Unclear risk (no information)	Low risk	Moderate risk	Moderate risk
Outcome: bleeding								
Triulzi et al. [34]	Moderate risk	Low risk	Low risk	Low risk	Low risk	Low risk	Moderate risk	Moderate risk
Outcome: CCI								
Kerkhoffs et al. [32]	Moderate risk	Low risk	Low risk	Low risk	Low risk	Low risk	Moderate risk	Moderate risk
Dijkstra-Tiekstra et al. [28]	Serious risk	Low risk	Low risk	Low risk	Moderate risk	Low risk	Moderate risk	Serious risk
Van Rhenen et al. [35]	Moderate risk	Low risk	Low risk	Low risk	Low risk	Low risk	Moderate risk	Moderate risk
Heuft et al. [33]	Serious risk	Low risk	Low risk	Low risk	Low risk	Moderate risk	Moderate risk	Serious risk
Slichter et al. [12]	Moderate risk	Low risk	Low risk	Low risk	Low risk	Low risk	Moderate risk	Moderate risk
Akkok et al. [31]	Moderate risk	Low risk	Low risk	Low risk	Low risk	Low risk	Moderate risk	Moderate risk
Heim et al. [36]	Moderate risk	Low risk	Low risk	Low risk	Unclear risk (no information)	Low risk	Moderate risk	Moderate risk
Dijkstra-Tiekstra et al. [29]	Moderate risk	Low risk	Low risk	Low risk	Low risk	Low risk	Moderate risk	Moderate risk
Outcome: PLT CI								
Kaplan et al., [30]	Serious	Low risk	Low risk	Low risk	Unclear risk	Low risk	Moderate risk	Serious risk
Outcome: TRAE								
Kaufman et al. [4]	Moderate risk	Low risk	Low risk	Low risk	Low risk	Low to moderate risk	Moderate risk	Moderate risk

*Abbreviations*: *CCI* corrected count increment, *PLT CI* platelet count increment, *TRAE* transfusion-related adverse events

#### Transfusion-related adverse events

Kaufman et al. [4], in a post hoc analysis of the Platelet Dose (PLADO) randomised controlled trial including 5034 prophylactic transfusions to 1102 stable haematology patients, did not find any association between PLT storage duration up to 5 days and TRAEs, including after adjustment for numerous confounders related to PLT product and patient characteristics [4, 37]. In a prospective study analysing 9923 transfusions, Heim et al. did not find any association between PLT storage time and TRAEs that included fever or erythema within 6 h of tranfusion, chills or urticarial within 2 h of tranfusion or anaphylaxis and sepsis related to transfusion [36]. Keeping with these findings, another study investigating this outcome did not find any difference in TRAEs between patients receiving fresh and old PLTs [30].

#### Bleeding events

Five studies investigated the impact of PLT storage on risk of bleeding, using variable definition criteria for bleeding, including the World Health Organisation severity scale [26, 27, 32–34]. Most of them did not find any association between stored PLT and bleeding risk. MacLennan et al. [26], in a two-centre randomised block crossover trial, compared the efficacy of 244 PLT transfusions stored for 2 to 5 versus 6–7 days in 122 stable haematology patients. Patients were randomised to an eight-block schedule, with each block consisting of both a 2- to 5-day-old and a 6- or 7-day-old PLT transfusion in random order. Transfused PLTs had a mean age of 3.8 days (± 1.0) in the 2–5 day-old PLT group compared to 6.4 (± 0.5) in the 6–7-day-old PLT group. The proportion of days with bleeding was the same in the 2–5-day-old PLT and the 6–7-day-old PLT groups when the first block was considered and when all evaluable blocks were considered (13.7% of days in the 2–5-day-old PLT group versus 12.2% of days in the 6–7-day-old PLT group in all evaluable blocks, *p* = 0.53). Although this study adjusted for confounders, a high proportion of screened patients were not eligible (51%) and bleeding was not the primary study outcome; therefore, it was not powered to detect a difference in bleeding [26].

In a post hoc analysis of a large randomised trial, PLT storage time did not have an impact on bleeding risk. Although the study had a large sample size, analysed data from a large randomised controlled trial and adjusted for many factors impacting on PLT transfusion efficacy, it excluded patients who were at higher risk of bleeding. Indeed, patients who experienced grade 2 or more bleeding before or on the day of the first PLT transfusion and those receiving multiple PLT units on the day of the first PLT transfusion were not eligible [34].

In a third study investigating the impact of PLT storage duration on prevention of bleeding, patients receiving PLT with storage duration of up to 4 days (median period of 53 h (interquartile range (IQR) 11–112)) had reduced need for RBC transfusion (38 versus 55%, *p* < 0.01) and less bleeding symptoms reported in the medical charts (22 versus 56%, *p* < 0.01) compared to those receiving PLT with a storage duration up to 5 days (median period of 78 h (IQR 11–135)). However, this study was a retrospective single-centre study and did not adjust for important confounders impacting on RBC requirement and bleeding events and differences in patient baseline characteristics between the two study periods [33]. In addition, there was a considerable overlap in PLT storage time between groups.

Kerkhoffs et al., in a randomised trial comparing outcomes after transfusion of PLTs stored in plasma and PLTs stored in platelet additive solution (PAS) in 278 patients, reported no association between storage time and bleeding [32].

Finally, in a single-centre randomised trial comparing transfusion outcomes in 60 haematology patients, PLT storage time was not associated with bleeding events. However, bleeding was not as clearly defined as in the other trials and the study was underpowered for this outcome [27].

In conclusion, there is no strong evidence to support an association between PLT storage duration and bleeding when PLT storage durations of 5 days or up to 7 days are considered. Nonetheless, the available studies exclude patients who are at highest risk of bleeding and did not address haemostatic efficacy in bleeding patients.

#### Platelet CI and time to next transfusion

CCI or CI are surrogate measures of transfused PLT survival in recipients and are commonly measured between 1 and 24 h post-PLT transfusion; however, variability in the timing of measurements makes comparisons between study findings difficult. Twelve of the 13 studies carried out in the haematology/oncology population analysed one of these transfusion outcomes. Ten studies reported an association between PLT storage duration and these outcomes [12, 27, 28, 30–36], one did not find any impact of PLT storage time on these outcomes [29], while one reported conflicting results depending on the statistical analysis performed [26] (Table 3).

In the randomised controlled trial conducted by MacLennan et al., the proportion of successful transfusions defined as an 8 to 24 h CCI of more than 4.5 was comparable when only the first evaluable randomisation block of 2- to 5-day-old and 6- or 7-day-old PLT transfusions per patient (*n* = 122 patients, *n* = 244 transfusions) was analysed [26].

In the post hoc analysis of the PLADO study described above, Triulzi et al. analysed 3447 transfusions and found that PLTs stored for 5 days were associated with a lower CCI and a lower absolute PLT count at 4 and 24 h, after adjustment for confounders, in comparison with PLTs stored for 3 days [34].

In a single-centre retrospective study evaluating the impact of reduced storage time of PLTs from 5 to 4 days, Heuft et al. [33] found that patients transfused with the freshest PLTs (median storage period of 53 h, IQR 11–112) had a higher CCI and a shorter time to the next PLT transfusion compared to those transfused with older PLTs (median storage period of 78 h, IQR 11–135). However, this study had a number of important limitations as mentioned previously and did not adjust for important confounders impacting on CCI [33].

In a prospective observational study of 67 haematology patients receiving prophylactic PLT transfusions, Dijkstra-Tiekstra et al. analysed the impact of prolonged storage duration (up to 7 days) on the rate of successful transfusion defined by CI at 1 h of at least 10 × 10^9^/L. The rates of successful transfusion between groups were similar (97% in both groups, *p* > 0.05). However, when compared with 7-day-old PLTs, transfusion with 2-day-old PLTs resulted in a significantly higher CI at 1 h (2-day-old PLT group, CI at 1 h = 34, 95% confidence interval 29.6–38.4 versus 28.7, 95% confidence interval 24.2–33.1, in the 7-day-old PLT group, *p* < 0.05) and significantly higher CCI (Table 3) [28].

Diedrich et al. [27], in a double blind randomised trial of 60 allogeneic stem cell transplant recipients prophylactically transfused with PLTs with storage durations of either 1–5 days or 6–7 days, found transfusion of fresher PLTs (2.9 days) was associated with a higher CCI at 1 and 24 h compared to older PLTs (6.6 days). Time to next transfusion was also longer in the 1–5-day-old PLT group than in the 6–7-day-old PLT group (Table 3). Study limitations included a small sample size and inclusion of only blood group O irradiated PLTs [27]. Keeping with these findings, Akkok et al. reported a significant decrease in 1- and 24-h CCI and in transfusion interval when PLT storage time increased in 77 haematology or oncology patients receiving 688 transfusions with PLTs stored for up to 6.5 days [31].

Slichter et al., in a post hoc analysis of the TRAP trial aiming to identify factors associated with post-transfusion platelet response and PLT refractoriness, found that PLTs stored for 48 h or less were associated with a higher CI at 1 and 18 to 24 h [12].

Van Rhenen et al. [35], in a trial including 103 patients comparing outcomes after transfusion of pooled buffy coat PLT components stored for up to 5 days with and without photochemical pathogen inactivation, found an independent association between 1-h CCI and PLT storage duration but not with 24-h CCI, bleeding or RBC transfusion volume. Although this was a randomised controlled trial, it was not designed to address the impact of PLT storage duration on outcomes [35]. The trial by Kerkhoff et al. reported similar results; however, it was also not designed to assess the effect of PLT storage duration [32].

In a multicentre observational study of 93 patients receiving one PLT unit per day, PLT storage time of up to 7 days did not impact CCI at 1 and 24 h. This study did not adjust for important confounders such as ABO mismatch [29]. Similarly, transfusion of PLTs stored for 6–7 days was not associated with changes in 24-h PLT CI compared to transfusion of PLTs stored for up 5 days in a retrospective study of 146 stable haematology patients [30].

Finally, Heim et al., in a prospective study of 672 haematology patients receiving mainly prophylactic PLT transfusion, reported that CCI was significantly and independently lower after transfusion of PLTs stored for 3 days or more [36].

In conclusion, of the 12 studies investigating associations between PLT storage duration and transfusion outcomes, ten found an association with CCI, CI or time to next transfusion. Shorter storage duration (less than 3 days) appears to be associated with an increase in CCI, CI and time to next transfusion, but not with rate of successful transfusion defined by a set PLT threshold.

## Discussion

In this systematic review, we found no strong evidence for an association between PLT storage duration and morbidity (including sepsis) or mortality in critically ill or haematology/oncology patients. There was also no evidence to support an association between PLT storage duration and bleeding when PLT storage duration of 5 days or up to 7 days is considered in stable haematology patients. However, the available literature in haematology patients suggests higher CCI following transfusion of PLTs stored for less than 3 days compared with 5- and 7-day-old PLTs.

Of note, studies investigating PLT storage duration and transfusion-centred outcomes have all been conducted in stable haematology patients. The effects of changes during PLT storage may not be deleterious when transfused to stable patients, but may have implications for patients who already have systemic inflammatory response syndrome, such as the critically ill. In addition, the haemostatic effect has not been assessed in actively bleeding patients to determine whether PLTs stored for prolonged periods are as effective. Prospective research investigating the efficacy and safety of stored PLTs is important as some countries already prolong PLT storage duration.

The effect of platelet storage time on transfusion or clinical outcomes has been studied in two other reviews and meta analyses [16, 17]. In keeping with our findings, the authors found that PLT storage time did affect platelet measurements. Nonetheless, there was no robust evidence to support a potential impact of PLT storage duration on patient-centred outcomes. Both meta-analyses used literature searches without any restriction on the year of publication, leading to a relatively high heterogeneity in PLT product analysed. The authors found that leucoreduction has led to a significant decrease in transfusion-related adverse effects, highlighting the importance to adjust for other parameters, including other PLT characteristics.

### Strengths and limitations

We performed a systematic review, following the PRISMA guidelines, examining a range of clinical- and transfusion-centred outcomes. We included studies in which the primary endpoint was not to study the impact of PLT storage duration on the outcome but which analysed PLT storage duration as a covariate.

Nonetheless, our systematic review suffers some limitations. Because of the variability in terms and the heterogeneity of the outcomes and settings, we were unable to perform any quantitative analysis. Our review is also limited by the quality of included studies, with the majority being observational, retrospective, with a relatively small sample size and not adjusting for key confounders. Findings of studies performed in stable patients cannot be generalisable to actively bleeding patients. Finally, we did not consider other PLT characteristics in the analysed outcomes; however, extensive information on PLT characteristics was unequally available in the included studies.

## Conclusions

We have only limited data on the impact of PLT storage duration on clinical outcomes in critically ill patients and none on the potential association between PLT storage duration and transfusion outcomes in these patients. Based on the results of studies conducted in haematology patients, prolonged platelet storage time (up to 5 to 7 days) seems to be associated with a decreased PLT CI and CCI compared to fresher PLTs (less than 3 days), while there is no evidence to support that PLTs stored up to 7 days are less effective on CCI than PLTs stored for 5 days. Based on the available literature in both haematology and critically ill patients, PLT storage time does not impact on the ability to prevent bleeding and transfusion of older PLTs (up to 5 days) is safe as found in large cohorts. Evidence is lacking and no conclusion can be drawn on the association between PLT storage time and the efficacy to stop active bleeding. There is insufficient evidence and no good quality studies to show that stored PLTs are as safe and effective as the freshest PLTs in massively transfused patients. Prospective research addressing these issues is warranted.

## Additional file

Additional file 1: Search strategy. (DOCX 68 kb)

## Abbreviations

CABG: Coronary artery bypass graft
CCI: Corrected count increment
CI: Count increment
ICU: Intensive care unit
IQR: Interquartile range
OR: Odds ratio
PAS: Platelet additive solution
PLT: Platelets unit
RBC: Red blood cell
TRAE: Transfusion-related adverse events
