# Supplementary material for: Platelet storage duration and its clinical and transfusion outcomes: a systematic review
Source: Crit Care. 2018 Aug 5;22:185. doi: 10.1186/s13054-018-2114-x (PMC6091146; doi:10.1186/s13054-018-2114-x)
Supplement: Supplementary file 1 — Search strategy. (DOCX 68 kb) [file 13054_2018_2114_MOESM1_ESM.docx]

**PubMed search of the following search terms restricted to human studies, English language papers only and papers published between January 2000 and July 2017:**

((platelet OR platelets OR "platelet transfusion")) AND ("blood storage" OR "blood preservation" OR "platelet storage" OR "shelf life" OR "storage duration") AND (outcome* OR mortality OR morbidity OR infection OR "count increment" OR "platelet count" OR "corrected count increment" OR bleed* OR "adverse event" OR "sepsis" OR bacteremia OR thrombocytopenia OR "treatment outcome" OR "treatment outcomes")

**MEDLINE, Embase and Cochrane databases via OVID of the following search terms restricted to human studies, English language papers only and papers published between January 2000 and July 2017:**

(platelet* OR platelet transfusion) AND (blood storage OR blood preservation OR shelf life OR storage duration) AND (outcome* OR mortality OR morbidity OR infection OR count increment OR platelet count OR corrected count increment OR hemorrhage OR thrombocytopenia OR bleed* OR adverse events OR sepsis OR bacteremia OR bacteraemia OR thrombocytopenia OR treatment outcome)
